# Whole cells of recombinant CYP153A6-*E. coli* as biocatalyst for regioselective hydroxylation of monoterpenes

**DOI:** 10.1186/s13568-022-01389-8

**Published:** 2022-04-27

**Authors:** Pietro Cannazza, Marco Rabuffetti, Silvia Donzella, Valerio De Vitis, Martina L. Contente, Maria da Conceição Ferreira de Oliveira, Marcos C. de Mattos, Francisco G. Barbosa, Ricardo Pinheiro de Souza Oliveira, Andrea Pinto, Francesco Molinari, Diego Romano

**Affiliations:** 1 Department of Food, Environmental and Nutritional Sciences (DeFENS), University of Milan, Via Mangiagalli 25, 20133 Milano, Italy; 2 Laboratory of Biotechnology and Organic Synthesis (LABS), Department of Organic and Inorganic Chemistry, Science Centre, Federal University of Ceará, Campus do Pici, Fortaleza, CE 60455-970 Brazil; 3 Department of Biochemical and Pharmaceutical Technology, Faculty of Pharmaceutical Sciences, University of São Paulo, São Paulo, SP Brazil

**Keywords:** Cytochrome P450, Hydroxylation, Monoterpene, Biocatalysis, Whole cells

## Abstract

Optimized recombinant whole cells of *E. coli* bearing CYP153A6 were employed for catalyzing the hydroxylation of different monoterpene derivatives. In most cases, high selectivity was observed with exclusive hydroxylation of the allylic methyl group bound to the aliphatic ring. In the case of (*R*)- and (*S*)-carvone, hydroxylation occurred also on the other allylic methyl group, although to a lesser extent. Biotransformations carried out in fed-batch mode on (*S*)-limonene and α-terpineol showed that recombinant whole cells retained activity for at least 24 h, allowing for the recovery of 3.25 mg mL^−1^ of (*S*)-perillyl alcohol and 5.45 mg mL^−1^ of 7-hydroxy-*α*-terpineol, respectively.

**Supplementary Information:**

The online version contains supplementary material available at 10.1186/s13568-022-01389-8.

## Keypoints

- Different monoterpenes can be regioselectively hydroxylated by CYP153A6 monooxygenase
- The biotransformation with whole cells is complementary to chemical oxyfunctionalization
- Fed-batch biotransformations have been applied for preparative purposes

**Supplementary Information:**

The online version contains supplementary material available at 10.1186/s13568-022-01389-8.

## Introduction

Allylic hydroxylation can be accomplished by a variety of chemical methods (especially by selenium and chromium reagents) with good chemo-, regio-, and stereoselectivity; however, catalytic reactions and the use of molecular oxygen as co-oxidant are strongly requested (Nakamura and Nakada 2013). Therefore, the use of transition metal catalysts able to use O_2_ has been developed as an alternative method for allylic C–H oxidation (Campbell and Stahl 2012). On the other hand, biocatalytic allylic hydroxylation offers a few advantages, such as mild reaction conditions and high selectivity, but they are often hampered by low productivity (Ortiz de Montellano 2010; Boeglin and Brash 2012; Bogazkaya et al. 2014). The efficiency can be limited by different factors, such as low (bio)catalyst stability, multi-component nature of the enzymatic system, and necessity for cofactors, as well as low substrate and O_2_ solubility (Julsing et al. 2008; Bernhardt and Urlacher 2014; Liang et al. 2018).

The cytochrome P450 CYP153 family is characterized by the ability to hydroxylate the terminal groups of *n*-alkanes (Maier et al. 2001). These enzymes have been found in different bacteria (Kubota et al. 2005), showing remarkable regioselectivity which has been especially exploited for α,ω-oxyfunctionalization of medium-chain alkanes (Pennec et al. 2014; Song et al. 2019; Park and Choi 2020).

Cytochrome P450 CYP153A6 from *Mycobacterium* sp. strain HXN-1500 is a soluble enzyme able to catalyze the selective hydroxylation of terminal methyl group of different alkanes (van Beilen et al. 2006). Whole cells (*Pseudomonas putida* or *Escherichia coli*) expressing heterologous CYP153A6 and its electron transport partners (ferredoxin reductase and ferredoxin) have been used as biocatalysts (Funhoff et al. 2006, 2007; Gudiminchi et al. 2012; Olaofe et al. 2013;); purified enzymes were also used separately to catalyze *n*-octane hydroxylation in vitro (Kochius et al. 2018). The enzyme showed no activity on methylene groups and very poor activity on simple cyclic alkanes (Funhoff et al. 2007). However, it was very active towards specific methyl groups, such as the terminal one of linear C_6_–C_11_ alkanes (thus furnishing the corresponding 1-alkanols) and the C7 one of (*S*)-limonene (giving (*S*)-perillyl alcohol) in high chemical purity (Funhoff et al. 2006, 2007; Olaofe et al. 2013).

Additionally, directed evolution of CYP153A6 allowed for hydroxylation of *n*-butane to 1-butanol (Koch et al. 2008). The use of CYP153A6 in whole cells is limited more by coupling efficiencies rather than cofactor supply (Pennec et al. 2014). Nevertheless, the most significant limitation in recombinant *E. coli* whole cells is hydrocarbon transport, with substrate import being the main determinant of hydroxylation rates, and product export playing a key role in the system stability (Cornelissen et al. 2013). Whole cell systems bearing co-expression of alkane transporters or systems for cofactor regeneration have been employed, together with two-liquid phase systems and permeabilization of the whole cells (Julsing et al. 2012). All these studies revealed that activity of CYP153A6 is characterized by high selectivity, allowing specific oxyfunctionalization of structurally different substrates.

In this work, starting from the observation of the selective hydroxylation of (*S*)-limonene, we have revisited the potential of CYP153A6 as preparative biocatalyst for the smooth hydroxylation of allylic methyl groups in twelve monoterpenes, thus strengthening the site-selective biocatalytic oxyfunctionalisation of limonene and other monoterpenes as a powerful tool to make added-value products from agrofood wastes (e.g. citrus waste) or to produce intermediates for subsequent (bio)conversions.

## Materials and methods

### Materials and chemicals

All reagents were purchased from Sigma-Aldrich (Milan, Italy) and/or from VWR International and were used without further purification. All the solvents were of HPLC grade. Analytical Thin Layer Chromatography TLC was performed on silica gel 60 F254 precoated aluminum sheets (0.2 mm layer; Merck, Darmstadt, Germany). Components were detected under an UV lamp (λ 254 nm), by spraying with a vanillin/H_2_SO_4_ solution in EtOH [6% (w/v) vanillin + 1% (v/v) H_2_SO_4_], followed by heating at about 150 °C. Product purification was accomplished by flash chromatography (silica gel 60, 40–63 mm, Merck).

### Prepararion of recombinant *E. coli* harbouring CYP153A6

The synthetic gene encoding CYP153A6 (BaseClear B.V., Leiden, The Netherlands) operon has been designed and amplified using the following primer:

Forward: 5′-CACCATATGACCGAAATGACCGTGGC-3′.

Reverse: 5′-ATTGCTCGAGTCAATGCTGCGCGGC-3′.

The amplified gene was then cloned into the pET100/D-TOPO® vector (Invitrogen) downstream the EK cleavage site, and correct construct sequence was confirmed by DNA sequencing (Eurofins Biolab Srl). The synthetic gene sequence has been deposited in NCBI database with accession number OM622424. Recombinant BL21(DE3)Star *E. coli* cells harbouring the pET100-CYP53A6 plasmid were obtained through heat-shock transformation.

### Expression of CYP153A6

Expression of the recombinant CYP153A6 operon was performed using BL21(DE3)Star *E. coli* strain harbouring pET100-CYP53A6 expression vector. Seed cultures were prepared inoculating 0.2 mL of glycerol stock of the recombinant strain in 20 mL of broth with 100 mg mL^−1^ ampicillin and incubated at 37 °C at 120 rpm in Erlenmeyer flasks for 16 h. The seed cultures were used as inoculum in 1 L baffled flasks containing 200 mL of the selected medium supplemented with ampicillin (100 mg mL^−1^) to get an initial cells density of 0.1 OD_600_. The resulting suspensions were incubated at 37 °C and 120 rpm until 0.6–0.8 OD_600_ (2–4 h), brought to 4 °C for 5 min and induced for 4 h with isopropyl-*β*-d-thiogalactopyranoside 0.5 mM at 28 °C and 150 rpm. The following liquid media were used: Luria–Bertani (LB: 10 g L^−1^ bacto-tryptone, 5 g L^−1^ yeast extract, 10 g L^−1^ NaCl), Super Broth (SB: 32 g L^−1^ bacto-tryptone, 20 g L^−1^ yeast extract and 5 g L^−1^ NaCl), Terrific Broth (TB: 12 g L^−1^ bacto-tryptone, 24 g L^−1^ yeast extract, 8 g L^−1^ glycerol, 9.4 g L^−1^ KH_2_PO_4_ and 2.2 g L^−1^ K_2_HPO_4_).

### Biotransformations

Cell growth was measured both as optical density (OD_600nm_, Eppendorf BioSpectrometer) than as cell dry weight (mg mL^−1^) of washed cells coming from a known volume of culture.

The enzymatic activity (Units) was calculated dividing the moles of substrate converted into product by the time unit (min) per weight of biocatalyst (U g^−1^ dry weight) or reaction volume (U L^−1^).

Optimization of biotransformations was carried out by varying different parameters of the reactions (pH, temperature, and biomass concentration) in sequential experimental trials selected by Multisimplex® 2.0 software. Cell pellets were recovered by centrifugation at 5000 rpm for 10 min at 4 °C, washed once with 100 mM potassium phosphate buffer pH 7.0 and suspended in different phosphate buffers to get the desired cells density; the suspensions were transferred to flat bottom baffled flasks without exceeding 10–15% of the total volume, and incubated at the desired temperature at 150 rpm. The substrates at different concentrations were added to the suspension and the flasks tightly sealed. To standardize the effect of volatility of 1a on the time course of the reaction, each flask was dedicated to a single analysis, thus avoiding repeated sampling.

Preparative biotransformations were carried out with 50 mg_dry cells_ mL^−1^ in a total volume of 50 mL of phosphate buffer (100 mM) at pH 8.0 at 28° C and 150 rpm. For GC analysis, proper amounts of the mixture (500 µL) were withdrawn at fixed times, extracted with EtOAc (1:1 volume ratio), dried under nitrogen stream at 4° C, diluted in EtOAc and directly injected. For product purification, the mixture was extracted with EtOAc (3 × reaction volume). The reunited organic phases were dried over anhydrous Na_2_SO_4_ and evaporated under vacuum at 4° C. The resulting crude material was purified by flash chromatography (*n*-hexane/EtOAc, from 5 to 45% EtOAc in *n*-hexane) to get either a mixture of constitutional isomers (**2c** + **3c** and **2d** + **3d**) or pure products (**2e**, **2f**, **2k** and **2l**). The ratio between isomers was determined by GC analysis.

### Analyses

GC analyses was performed using a *Dani® 86.10 HT* gas chromatographer equipped with a flame ionization detector (200 °C, p(H_2_) 0.8 atm, p(air) 1.5 atm). Chromatographic conditions were as follows: column, DeMePeβCDxPS086 Mega® (25 m × 0.25 mm); injection volume: 1 µL (split (1/50), 230 °C); injection solvent: EtOAc; carrier: H_2_ (0.6 mL/min). Analyses were performed with the following program: (i) gradient from 80 °C to 110 °C (10 °C/min), (ii) isocratic at 110 °C for 9 min. Data were processed with the *EZChrom Elite* software. Retention times were reported in minutes. ^1^H NMR spectra were recorded on a Varian Oxford 300 MHz NMR spectrometer equipped with a *VnmrJ* software package (Varian Medical Systems, Palo Alto, California, USA) at 300 K, unless stated otherwise. ^1^H chemical shifts (*δ*) are given in parts per million and were referenced to the solvent signals (*δ*_H_ 7.26 ppm from tetramethylsilane (TMS) for CDCl_3_).

### Chemical synthesis of substrates

Synthesis of **1e** and **1f**: LiAlH_4_ (1.0 M in THF, 4.0 mL, 4.00 mmol, 1.20 equiv) was added dropwise at -78° C to a solution of **1c** or **1d** (0.52 mL, 3.32 mmol, 1.00 equiv) in dry THF (10 mL) under inert atmosphere. The mixture was stirred while warming to room temperature for 3 h. Water (1 mL), 2 M NaOH (2 mL) were added at 0° C and the suspension was extracted with Et_2_O (3 × 20 mL). The reunited organic phases were then washed with brine (2 × 10 mL), dried over anhydrous Na_2_SO_4_ and evaporated. The resulting crude material was purified by flash column chromatography (cyclohexane-EtOAC, 85:15). Compound **1e** was obtained as a colorless oil in quantitative yield (504 mg, 3.31 mmol): ^1^H NMR (300 MHz, CDCl_3_): *δ* 5.53–5.48 (m, 1H, H^1^), 4.75–4.73 (m, 2H, CH_2_^9^), 4.24–4.15 (m, 1H, H^3^), 2.33–2.22 (m, 1H, H^4/5/6^), 2.21–2.12 (m, 1H, H^4/5/6^), 2.08–2.02 (m, 1H, H^4/5/6^), 2.02–1.88 (m, 1H, H^4/5/6^), 1.76 (dt, *J* = 4.0, 1.5 Hz, 3H, CH_3_^7/10^), 1.75 (br t, *J* = 1.1 Hz, 3H, CH_3_^7/10^), 1.51 (td, *J* = 12.1, 9.5 Hz, 1H, H^4/5/6^, partially covered by H_2_O) (Elamparuthi et al. 2012). Compound **1f** was obtained as a colorless oil in 99% yield (502 mg, 3.30 mmol): ^1^H NMR (300 MHz, CDCl_3_): *δ* 5.53–5.48 (m, 1H, H^1^), 4.75–4.73 (m, 2H, CH_2_^9^), 4.24–4.15 (m, 1H, H^3^), 2.33–2.22 (m, 1H, H^4/5/6^), 2.21–2.12 (m, 1H, H^4/5/6^), 2.08–2.02 (m, 1H, H^4/5/6^), 2.02–1.88 (m, 1H, H^4/5/6^), 1.76 (dt, *J* = 4.0, 1.5 Hz, 3H, CH_3_^7/10^), 1.75 (br t, *J* = 1.1 Hz, 3H, CH_3_^7/10^), 1.51 (td, *J* = 12.1, 9.5 Hz, 1H, H^4/5/6^, partially covered by H_2_O) (Elamparuthi et al. 2012).

Synthesis of **1g** and **1h**: pyridine (151 µL, 1.87 mmol, 1.89 equiv) and acetyl chloride (1.50 equiv) were added at 0 °C to a solution of **1e** or **1f** (150 mg, 0.99 mmol, 1.00 equiv) in dry CH_2_Cl_2_ (2.0 mL) under N_2_. The mixture was stirred at room temperature for 5 h. The resulting yellow suspension was diluted with CH_2_Cl_2_ (30 mL) and washed with sat. NH_4_Cl (2 × 30 mL), followed by sat. NaHCO_3_ (2 × 30 mL). The organic phase was dried over anhydrous Na_2_SO_4_ and evaporated. The resulting crude was purified by flash column chromatography (*n*-hexane–EtOAc, 7:3). **1g** was obtained as a colorless oil in 43% yield (82 mg, 0.42 mmol); **1h** was obtained as a colorless oil in 42% yield (86 mg, 0.41 mmol): ^1^H NMR (300 MHz, CDCl_3_): *δ* 5.61 (dt, *J* = 5.2, 3.4, 1.7 Hz, 1H, H^1^), 5.50–5.40 (m, 1H, H^3^), 4.76–4.66 (m, 2H, CH_2_^9^), 2.37–2.25 (m, 1H, H^4/5/6^), 2.24–2.15 (m, 1H, H^4/5/6^), 2.09–2.03 (m, 4H, CH_3_^12^, H^4/5/6^), 2.02–1.88 (m, 1H, H^4/5/6^), 1.73–1.71 (m, 3H, CH_3_^7/10^), 1.64 (td, *J* = 2.5, 1.4 Hz, 3H, CH_3_^7/10^), 1.49 (ddd, *J* = 13.0, 11.9, 10.0 Hz, 1H, H^4/5/6^) (Trost and Schmuff 1985).

Synthesis of **1i** and **1j**: pyridine (302 µL, 3.74 mmol, 3.78 equiv) and benzoic anhydride (335 mg, 1.48 mmol, 1.50 equiv) were added to a solution of **1e** or **1f** (150 mg, 0.99 mmol, 1.00 equiv) in EtOAc (2.0 mL) under N_2_. The mixture was refluxed overnight. The light orange solution was washed with sat. NH_4_Cl (2 × 30 mL) and sat. NaHCO_3_ (2 × 30 mL), dried over Na_2_SO_4_ and evaporated. The resulting crude was purified by flash column chromatography (*n*-hexane–EtOAc, 8:2) to get compounds **1i** and **1j** as colorless oils (**1i**: 33 mg, 0.13 mmol, 13%; **1j**: 38 mg. 0.15 mmol, 15%): ^1^H NMR (300 MHz, CDCl_3_): *δ* 8.10–8.04 (m, 2H, Ph), 7.60–7.53 (m, 1H, Ph), 7.48–7.41 (m, 2H, Ph), 5.76–5.63 (m, 2H, H^1^, H^3^), 4.77–4.70 (m, 2H, CH_2_^9^), 2.46–2.28 (m, 2H, 2 × H^4/5/6^), 2.22–1.94 (m, 2H, 2 × H^4/5/6^), 1.74 (br t, 3H, *J* = 1.1 Hz, CH_3_^7/10^), 1.71 (tq, *J* = 2.4, 1.1 Hz, 3H, CH_3_^7/10^), 1.67–1.61 (m, 1H, H^4/5/6^) (Correia and DeShong 2001).

7-Hydroxy-(*R*)-carvone and 10-hydroxy-(*R*)-carvone (**2c** + **3c**). The product mixture was obtained as an off-white solid in 67% yield. ^1^H NMR (300 MHz, CDCl_3_): *major isomer* (**2c**): *δ* 6.97–6.90 (m, 1H, H^1^), 4.82–4.79 (m, 1H, CH^9a^), 4.76–4.73 (m, 1H, CH^9b^), 4.24 (br s, 2H, CH_2_^7^), 2.77–2.25 (m, 5H, CH_2_^4^, CH_2_^6^, H^5^), 1.74 (s, 3H, CH_3_^10^); *minor isomer* (**3c**): *δ* 6.97–6.90 (m, 1H, H^1^, covered by **2c**), 5.11–5.08 (m, 1H, CH^9a^), 4.93–4.91 (m, 1H, CH^9b^), 4.11 (br s, 2H, CH_2_^10^), 2.77–2.25 (m, 5H, CH_2_^4^, CH_2_^6^, H^5^, covered by **2c**), 1.74 (s, 3H, CH_3_^7^, covered by **2c**) (Lakshmi et al. 2005).

7-Hydroxy-(*S*)-carvone and 10-hydroxy-(*S*)-carvone (**2d** + **3d**). The product mixture was obtained as an off-white solid in 70% yield. ^1^H NMR (300 MHz, CDCl_3_): *major isomer* (**2d**): *δ* 6.97–6.92 (m, 1H, H^1^), 4.85–4.80 (m, 1H, CH^9a^), 4.79–4.75 (m, 1H, CH^9b^), 4.30–4.23 (br s, 2H, CH_2_^7^), 2.79–2.27 (m, 5H, CH_2_^4^, CH_2_^6^, H^5^), 1.76 (s, 3H, CH_3_^10^); *minor isomer* (**3d**): *δ* 6.97–6.92 (m, 1H, H^1^, covered by **2d**), 5.12 (br s, 1H, CH^9a^), 4.95 (br s, 1H, CH^9b^), 4.16–4.08 (m, 2H, CH_2_^10^), 2.79–2.27 (m, 5H, CH_2_^4^, CH_2_^6^, H^5^, covered by **2d**), 1.76 (s, 3H, CH_3_^7^, covered by **2d**) (Lakshmi et al. 2005).

(4*R*,6*R*)-7-Hydroxycarveol (**2e**). The product was obtained as an off-white solid in 68% yield. ^1^H NMR (300 MHz, CDCl_3_): *δ* 5.77 (br s, 1H, H^1^), 4.75 (s, 2H, CH_2_^7^), 4.57–4.47 (m, 1H, H^3^), 4.23 (s, 2H, CH_2_^7^), 2.56 (br s, 2H, 2 × OH), 2.35–2.22 (m, 1H, CH_2_^4^/CH_2_^6^/CH^5^), 2.21–2.09 (m, 2H, CH_2_^4^/CH_2_^6^/CH^5^), 2.09–1.94 (m, 1H, CH_2_^4^/CH_2_^6^/CH^5^), 1.74 (s, 3H, CH_3_^10^), 1.58 (td, *J* = 12.1, 10.0 Hz, 1H, CH_2_^4^/CH_2_^6^/CH^5^) (Gimalova et al. 2012).

(4*S*,6*S*)-7-Hydroxycarveol (**2f**). The product was obtained as an off-white solid in 64% yield. ^1^H NMR (300 MHz, CDCl_3_): *δ* 5.76 (br s, 1H, H^1^), 4.74 (s, 2H, CH_2_^7^), 4.56–4.45 (m, 1H, H^3^), 4.21 (s, 2H, CH_2_^7^), 2.99–2.57 (m, 2H, 2 × OH), 2.34–2.21 (m, 1H, CH_2_^4^/CH_2_^6^/CH^5^), 2.20–2.08 (m, 2H, 2 × CH_2_^4^/CH_2_^6^/CH^5^), 2.07–1.92 (m, 1H, CH_2_^4^/CH_2_^6^/CH^5^), 1.73 (s, 3H, CH_3_^10^), 1.56 (q, *J* = 12.0 Hz, 1H, CH_2_^4^/CH_2_^6^/CH^5^) (Gimalova et al. 2012).

δ-3-Caren-10-ol (**2k**). The product was obtained as an oil in 39% yield. ^1^H NMR (300 MHz, CDCl_3_): *δ*

5.72–5.49 (m, 1H, H^4^), 3.92 (s, 2H, CH_2_^10^), 2.55–2.32 (m, 2H, CH_2_^2^/CH_2_^5^), 2.08–1.95 (m, 2H, CH_2_^2^/CH_2_^5^), 1.63 (s, 1H, OH), 1.11 (s, 3H, CH_3_^8/9^), 0.82 (s, 3H CH_3_^8/9^), 0.74–0.81 (m, 1H, H^1/6^), 0.69 (br t, *J* = 8.4 Hz, 1H, H^1/6^) (Frąckowiak et al. 2006).

7-Hydroxy-*α*-terpineol (**2l**). The product was obtained as an off-white solid in 65% yield. ^1^H NMR (300 MHz, CDCl_3_): *δ* 5.71 (br s, 1H, H^1^), 4.07–3.97 (m, 2H, CH_2_^7^), 2.22–2.02 (m, 3H, CH_2_^3^/CH_2_^4^/ CH_2_^6^/CH^5^), 2.01–1.94 (m, 1H, CH_2_^3^/CH_2_^4^/CH_2_^6^/CH^5^), 1.93–1.82 (m, 1H, CH_2_^3^/CH_2_^4^/CH_2_^6^/CH^5^), 1.61–1.51 (m, 1H, CH_2_^3^/CH_2_^4^/CH_2_^6^/CH^5^), 1.34–1.24 (m, 1H, CH_2_^3^/CH_2_^4^/CH_2_^6^/CH^5^), 1.22 (s, 3H, CH_3_^9^), 1.21 (s, 3H, CH_3_^10^) (Constantino et al. 2007).

## Results

### Optimization of microbial growth and activity

**Fig. 1:**
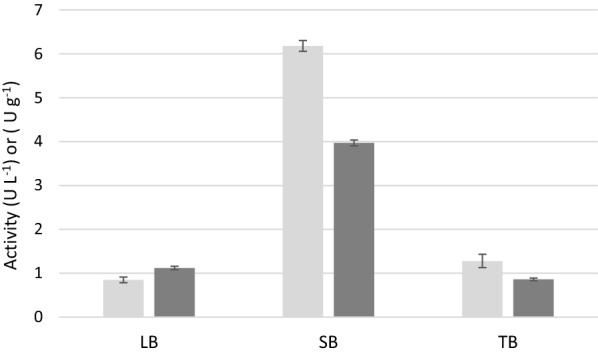
Activity of CYP153A6 towards (*S*)-limonene (2 mM) in *E. coli* using different media, expressed as U L^−1^ (dark grey bars) and U g^−1^_dry weight_ (light grey bars) after induction with 0.5 mM IPTG at mid-exponential phase (cells collected after 16 h at 28° C)

Chemically competent cells of *E. coli* BL21 Star™ (DE3) were transformed by expression of the redox synthetic gene operon (CYP153A6) which encodes a cytochrome P450, a ferredoxin, and a ferredoxin reductase from *Mycobacterium* sp. strain HXN-1500, by using the broad-host-range vector pET100. Growth of *E. coli* and expression of CYP153A6 were optimized by using three culture media (LB, SB and TB broth) and different times of growth and induction (0.5–1 mM IPTG). The observed specific growth rates were higher in SB (0.45 h^−1^) than in LB and TB (0.38 h^−1^ and 0.31 h^−1^, respectively); SB media allowed higher production of biomass (2.9 g L^−1^) in comparison to LB (0.6 g L^−1^) and TB (1.8 g L^−1^). In addition, SB medium gave higher enzymatic activity towards (*S*)-limonene (**1a**): up to fourfold increases were observed in SB medium, corresponding to a volumetric activity of 3.87 ± 0.07 U L^−1^ and specific activity of 6.18 ± 0.12 U g_dry cells_^−1^ (Fig. 1).

### Optimization of biotransformation with whole recombinant cells

**Fig. 2:**
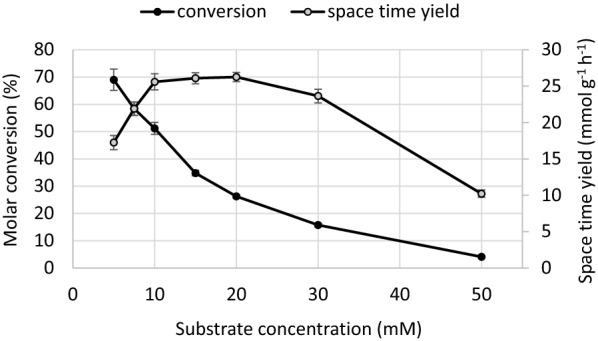
Effect of substrate concentration on the biotransformation of (*S*)-limonene with recombinant *E. coli.* Molar conversion (black circles) and space–time yields (grey circles) after 4 h

Resting cells of *E. coli* BL21 Star™ (DE3) bearing the redox synthetic gene operon (CYP153A6) and grown in SB liquid medium were employed for the hydroxylation of (*S*)-limonene (**1a**) in different phosphate buffers. Optimization was firstly performed by keeping the concentration of substrate constant (7.5 mM) and by simultaneously evaluating different parameters of the biotransformation (pH 6, 6.5, 7.0, 7.5, 8.0), temperature (25 °C, 28 °C, 30 °C, 37 °C), and biomass concentration (10, 20, 30, 40, 50 mg_dry cells_ mL^−1^), using a Multisimplex experimental design (Romano et al. 2011). Formation of perillyl alcohol **2a** ended after 4 h and no side-product was observed. Though the experimental setup was designed in order to minimize the effect of the immiscibility and high volatility of **1a** on the accurate measurement of the molar conversion, space–time yield (expressed as amount of product obtained after 4 h per gram of dry cell) was chosen as response parameter. Optimized conditions were found at relatively high cell density (50 mg_dry cells_ mL^−1^) in phosphate buffer 100 mM pH 8.0 and 28 °C. This allowed the formation of 0.66 mg mL^−1^ of (*S*)-perillyl alcohol after 4 h, corresponding to a space–time yield of 21.7 μmol_product_ g_cells_^−1^ h^−1^. Thereafter, the effect of substrate concentration was investigated using these optimized conditions (Fig. 2).

The best compromise between conversion and space–time yield was found at an initial substrate concentration of 10 mM (Fig. 2). The use of water-miscible organic solvents (ethanol, DMSO, DMF, acetone) for enhancing the solubility of **1a** did not allow noticeable improvement of the space–time yield. Interestingly, only traces (< 5%) of perillaldehyde were also detected at prolonged times, implying a negligible activity of unspecific oxidative enzymes of the whole cells system towards **2a**.

It is worth noting that very similar space–time yield (20.23 μmol_product_ g_cells_^−1^ h^−1^) was observed when (*R*)-limonene **1b** was used as substrate, showing that the stereocenter at C-6 position does not affect the enzyme activity.

### Biotransformations of other monoterpene derivatives

**Fig. 3:**
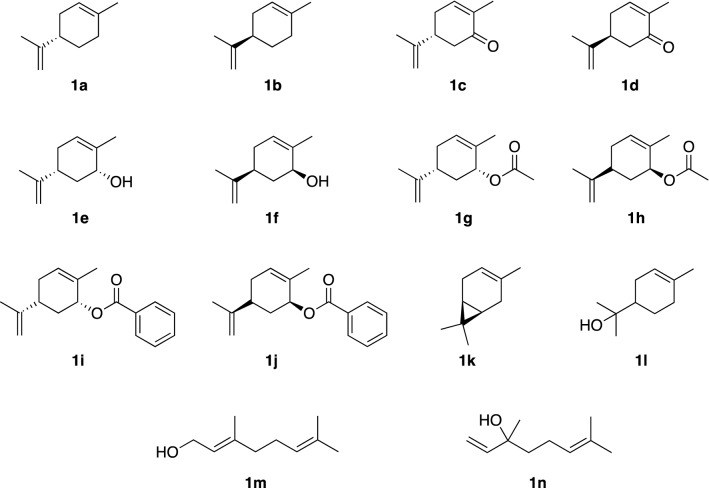
Panel of substrates tested for hydroxylation catalyzed by CYP153A6

Recombinant whole cells of *E. coli*, grown under optimized conditions, were used as resting cells for the biotransformation of the monoterpene derivatives **1c**–**1n** (Fig. 3).

Hydroxylation of (*R*)-carvone (**1c**) was firstly tested at 10 mM substrate concentration with total substrate consumption, yielding two regioisomers (**2c** and **3c**), resulted from hydroxylation at C7 and C10, respectively (entry 4, Table 1). Hydroxylation at C-7 was preferred, although the observed regioselectivity was limited (57/43 ratio between the two regioisomers). In this case, recovered yields were higher than the ones obtained with limonene, most likely because **1c** and its hydroxylation products are less volatile. The effect of **1c** concentration on the regioselectivity of the enzyme was also investigated (Table 1).

**Table 1:** Effect of substrate concentration in the hydroxylation of (*R*)-carvone **1c** using whole recombinant cells of *E. coli* harboring CYP153A6 expressed as molar conversion after 5 h


Entry	Substrate concentration (mM)	Conversion (%)^a^	**2c**/**3c** ^b^
1	2.5	78	87/13
2	5.0	75	77/23
3	7.5	74	66/34
4	10.0	72	57/43

^a^Calculated as amounts of total products recovered per amount of substrate

^b^Determined by gas-chromatography

At low substrate concentration (2.5 mM), the formation of **2c** (87/13 ratio of **2c**/**3c**) was markedly predominant (entry 1, Table 1). When substrate concentration was increased, the conversion remained in the range of 72–75%, but with higher production of the regioisomer **3c**, indicating a substantial competition between the two possible allylic hydroxylations.

Hydroxylation of other terpene derivatives catalyzed by CYP153A6 is displayed in Table 2. The reaction occurred on (*S*)-carvone (**1d**) with selectivity and conversions similar to those observed for the *R*-enantiomer, showing again that activity was not affected by the stereochemistry of the substrate.

**Table 2:** Hydroxylation of monoterpene derivatives using recombinant cells of *E. coli* harboring CYP153A6

Entry	Substrate	Product	Recovered yield (%)
1	**1d**	**2d**	72
2	**1e**	**2e**	68
3	**1f**	**2f**	64
4	**1g**	**2e**	65
5	**1h**	**2f**	69
6	**1i**	No reaction	–
7	**1j**	No reaction	–
8	**1k**	**2k**	39
9	**1l**	**2l**	65
10	**1m**	No reaction	–
11	**1n**	No reaction	–

Biotransformation conditions: substrates (10.0 mM) were added to the suspension of whole cells of recombinant *E. coli* (50 mg mL^−1^) in phosphate buffer (100 mM, pH 8.0) at 28° C

Products were recovered after 5 h of biotransformation

The regioselectivity of CYP153A6 was further investigated using the two *syn*-stereoisomers of carveol (**1e**: *R,R*-stereoisomer; **1f**: *S,S*-stereoisomer) as substrates. These compounds were transformed with total regioselectivity (hydroxylation in C7 position), furnishing diols **2e** and **2f**, respectively (entries 2 and 3, Table 2). Biotransformation of the enantiomers of carveol acetate (**1g** and **1h**) resulted in the formation of diols **2e** and **2f**, revealing that hydroxylation proceeded together with acetate hydrolysis: the latter was catalyzed by unspecific endogenous esterase(s) present in the whole cells (BL21DE3Star* E. coli* cells transformed with the empty vector showed hydrolysis of **1g** and **1h**, while no hydrolysis was observed in the absence of biocatalyst). Benzoyl esters of carveol **1i** and **1j** were not converted at all, showing that hydroxylation cannot occur on these bulkier substrates. Moreover, no endogenous esterase(s) of *E. coli* hydrolyzed the benzoyl ester.

In our screening for studying the substrate scope of CYP153A6, we included ∆^3^-carene (**1k**) and α-terpineol (**1l**). Selective hydroxylation of the allylic methyl group was found as the only apparent reaction in both substrates, yielding products **2k** (39%) and **2l** (65%), respectively. No other by-products were observed either by GC or during the isolation of the products, indicating that substrate volatility limited the real conversion (as in the case of limonene). Finally, no activity was observed on acyclic monoterpenes, geraniol (**1m**) and linalool (**1n**), encompassing allylic methyl groups (entries 10 and 11, Table 2).

### Fed-batch biotransformation

Fed-batch biotransformation of (*S*)-limonene (**1a**) was carried out for improving the amounts of product accumulated during the bioprocess. Fresh substrate **1a** (10 mM) was added after 4 h of biotransformation, when no residual substrate was present.

**Fig. 4:**
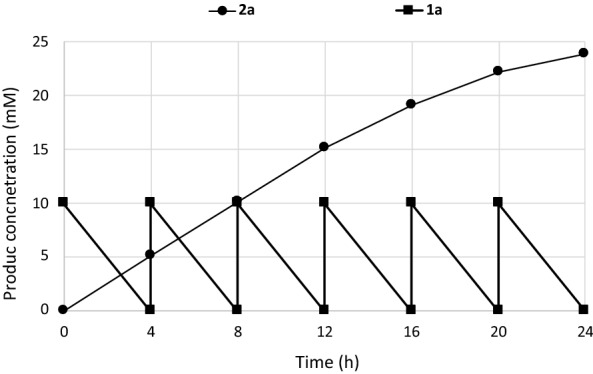
Fed-batch biotransformation of (*S*)-limonene with recombinant cells of *E. coli* harboring CYP153A6

Whole cells progressively lost hydroxylating activity and, after 24 h of fed-batch operation, 30–35% of the original activity was maintained; the reaction occurred with minor accumulation of aldehyde (< 0.1 mg/mL). After 24 h of operation, 23.9 mM (3.25 mg mL^−1^) of **2a** were accumulated in the biotransformation medium (Fig. 4).

A similar procedure was also applied, as proof of concept, to the hydroxylation of α-terpineol furnishing 32.0 mM (5.45 mg mL^−1^) of the corresponding hydroxylated product **2l**.

## Discussion

Cytochrome P450 CYP153 is an enzyme family known for the biocatalytic terminal hydroxylation of different types of molecules (Liang et al. 2018). Functionalization of methyl groups under mild conditions is an attractive biotransformation since it is difficult to perform with green methodology and high selectivity using conventional synthetic methods (Nakamura and Nakada 2013; Campbell and Stahl 2012). Cytochrome P450 CYP153A6 from *Mycobacterium* sp. strain HXN-1500 was previously found as a selective system for the hydroxylation of methyl groups contained in linear and cyclic hydrocarbons, such as *n*-octane, (*S*)-limonene, and *p*-cymene (Pennec et al. 2014; Cornelissen et al. 2013). The entire operon of CYP153A6 consists of the monooxygenase and its electron partners (ferredoxin reductase and ferredoxin) and their recombinant expression was achieved in different bacterial hosts (*P. putida*, *E. coli*); a noteworthy improvement of the biocatalyst activity was obtained by expressing the operon with a pET vector *in E. coli* (Gudiminchi et al. 2012). In this work, we used a similar vector and the activity towards (*S*)-limonene was optimized by studying different growth media, noticing that Super Broth liquid medium (SB) was particularly suited for promoting the desired activity.

The selectivity displayed towards (*S*)-limonene is remarkable, since CYP153A6 distinguishes between the two methyl groups both in allylic position and hydroxylates only the one directly attached to the aliphatic ring (Cornelissen et al. 2013). In this study we investigated whether this selectivity could be exploited for the hydroxylation of a series of monoterpene derivatives carrying allylic methyl groups. Complete selectivity for the hydroxylation of the methyl group directly attached to the ring (C7 for menthane structure and C10 for carane structure) was observed in most of the cases, with the exception of carvone that was also hydroxylated at the allylic methyl group at C10. The presence of a substituent at C6 position seems relevant for the recognition of the substrate. The two enantiomers of *cis*-carveol (with OH at C6 position) were regioselectively hydroxylated on the C7 methyl, with no trace of other products, and the same situation was observed with no substituents at C6; on the other hand, the two enantiomers of carvone (with a C = O at C6 position) were hydroxylated at both allylic positions, indicating that the presence of the carbonyl group partially hampered reactivity at C7.

Preparative biotransformations (50 mL-scale) were accomplished starting from 10 mM substrates in variable yields (0.6–1.1 mg mL^−1^), strongly depending on the volatility of the compounds involved in the reactions; product accumulation was improved with a simple fed-batch procedure. The fed-batch process was applied to the hydroxylation of (*S*)-limonene and α-terpineol (chosen for their different volatility and as general concept validation), allowing for the recovery of 3.25 mg mL^−1^ of (*S*)-perillyl alcohol **2a** and 5.45 mg mL^−1^ of 7-hydroxy-α-terpineol **2k**. In both cases, the biocatalyst maintained good activity for 16 h and lost around 65–70% of the initial activity after 24 h.

Optimized whole cells of *E. coli* harboring the operon of monooxygenase CYP153A6 have been used for highly regioselective hydroxylation of different monoterpene derivatives. Hydroxylation predominantly occurred at the allylic methyl group attached to the ring, even in the presence of other allylic methyl groups, indicating a fine selectivity. Notably, this selectivity is complementary to the one normally observed with chemical reagents used for allylic hydroxylations (Nakamura and Nakada 2013). Therefore, this research provides an alternative solution for the selective oxyfunctionalization of monoterpene derivatives, and more generally, paves the way to the modification of natural substrates into derivatives with higher hydrophilicity and lower volatility using a convenient microbial recombinant system.

## Supplementary Information

**Additional file 1:** 1. GC analyses. 2. SDS-PAGE.

## Data Availability

All data generated or analysed during this study are included in this published article and in the Additional file 1.
