# Supplementary material for: Whole cells of recombinant CYP153A6-E. coli as biocatalyst for regioselective hydroxylation of monoterpenes
Source: AMB Express. 2022 Apr 27;12:48. doi: 10.1186/s13568-022-01389-8 (PMC9046528; doi:10.1186/s13568-022-01389-8)
Supplement: Supplementary file 1 — Additional file 1: 1. GC analyses. 2. SDS-PAGE. [file 13568_2022_1389_MOESM1_ESM.docx]

**Whole cells of recombinant CYP153A6-*E. coli* as biocatalyst for regioselective hydroxylation of monoterpenes**

**SUPPORTING INFORMATION**

1. **GC ANALYSES**


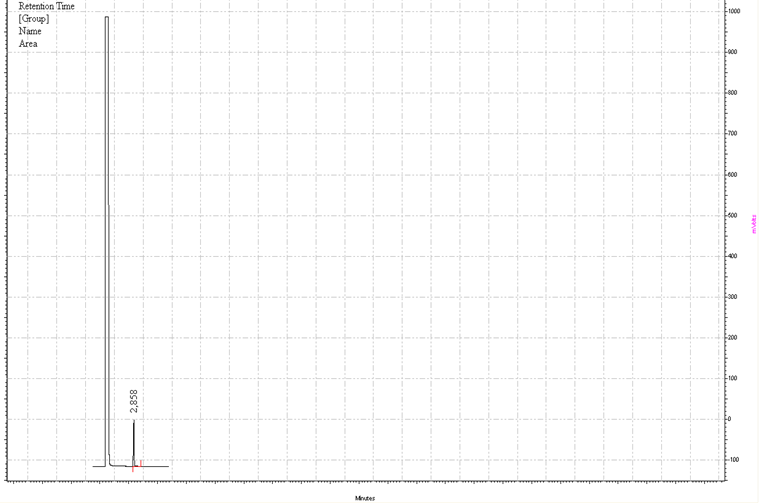


***S-*limonene (1a) (2,858 min)**

**)**


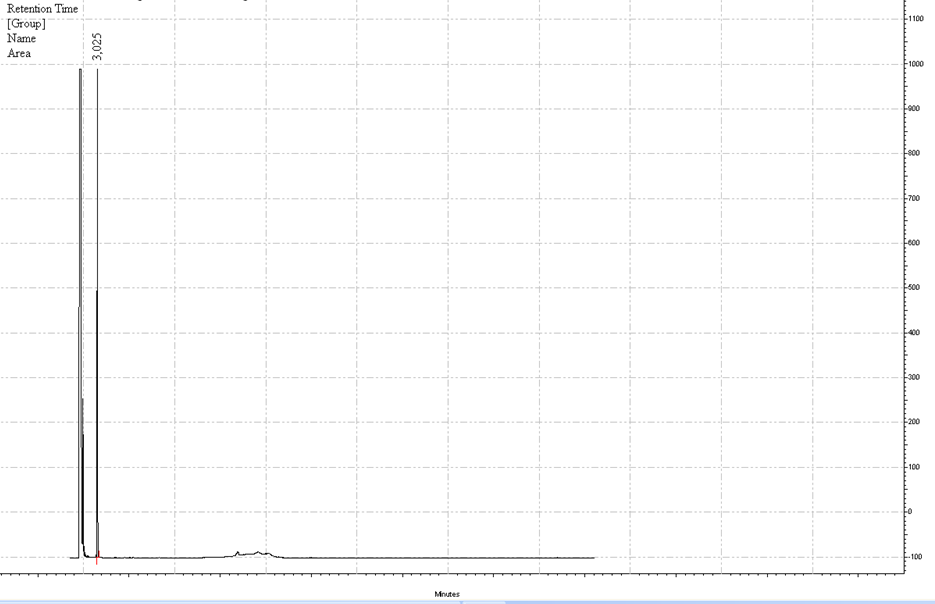


***R-*limonene (1b) (3,025 min)**

**)**


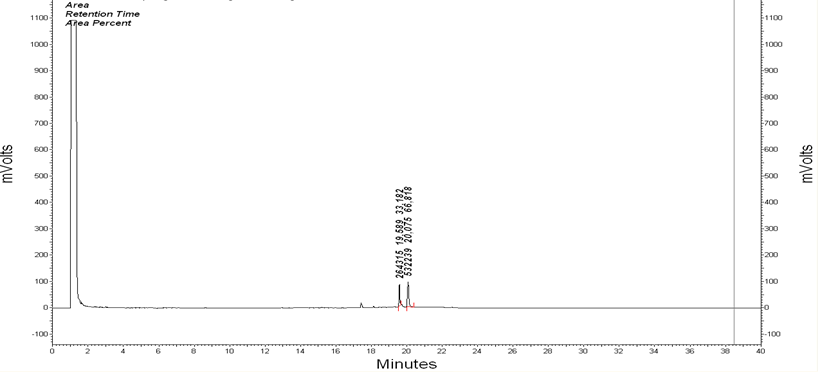


***S* – hydroxycarvone (2d – 3d) (19,58min – 20,07min)**


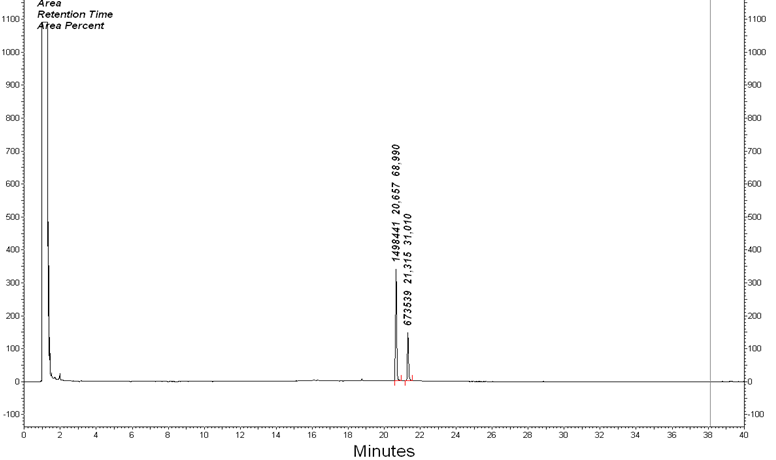


***R* – Hydroxycarvone (2c -3c) (20,65min – 31,01min)**


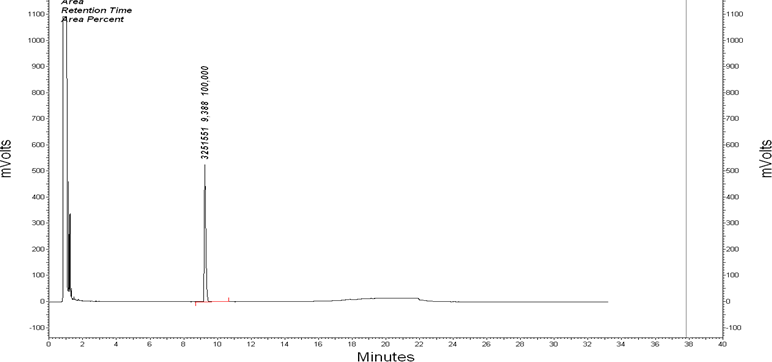


***R* - carvone (1c) (9,38min)**


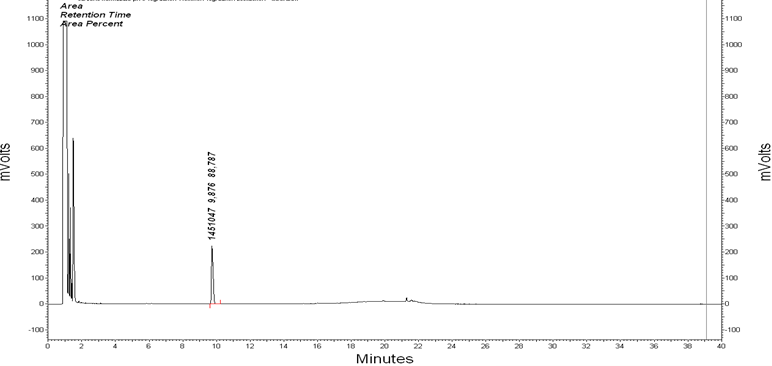


***S* – carvone (1d) (9,78min)**


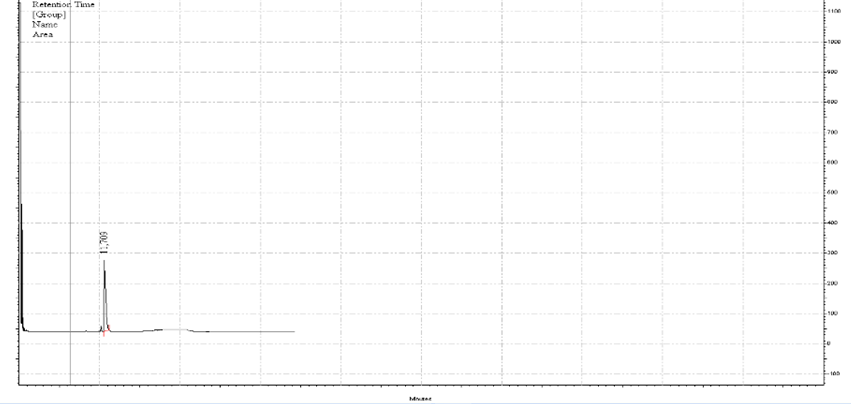


***S* – carveol (1f) (11,70min)**


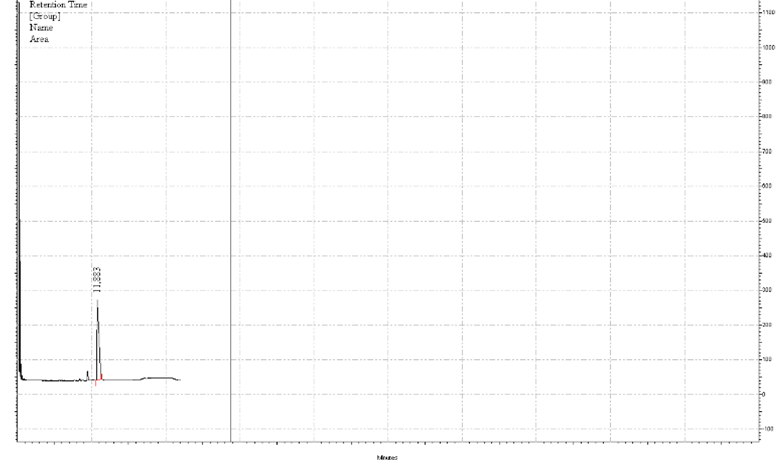


***R* – carveol (1e) (11,88min)**


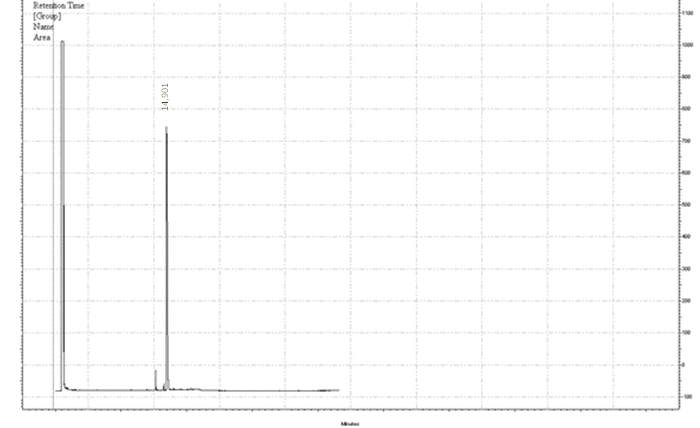


***R* - perillyl alcohol (2b) (14,90min)**

)


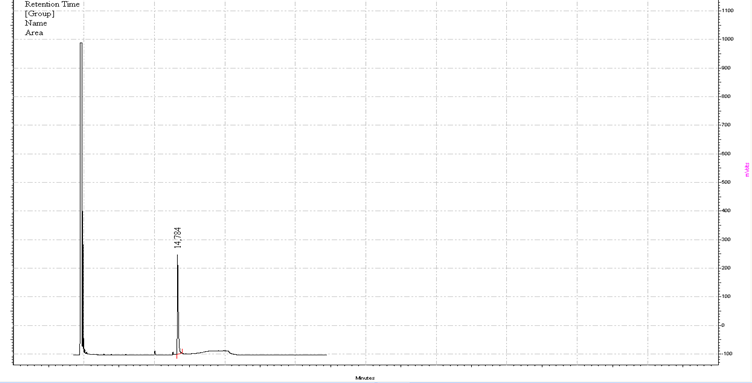


***S* - peryllil alcohol (2a) (14,78min)**


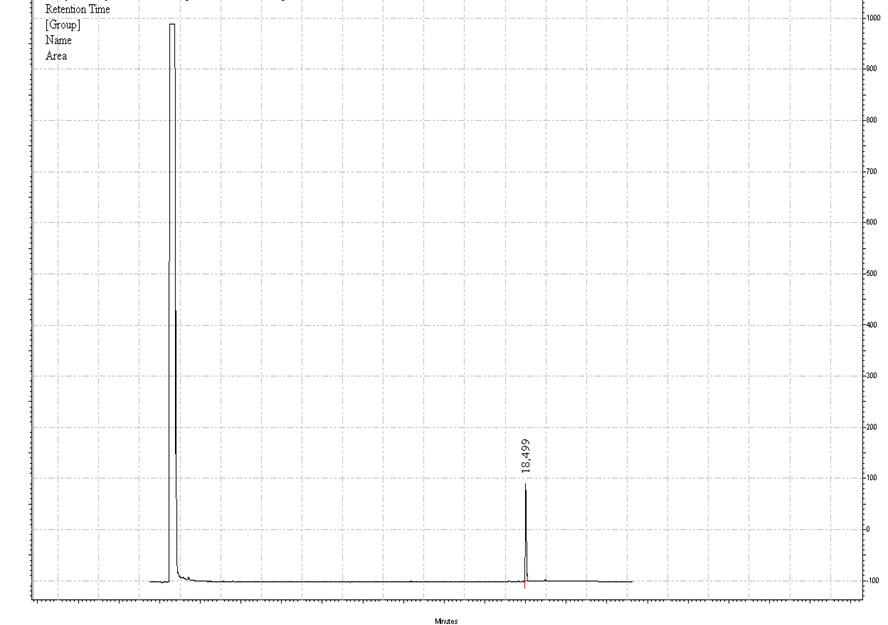


***R* - benzoyl carveol (1i) (14,49min)**


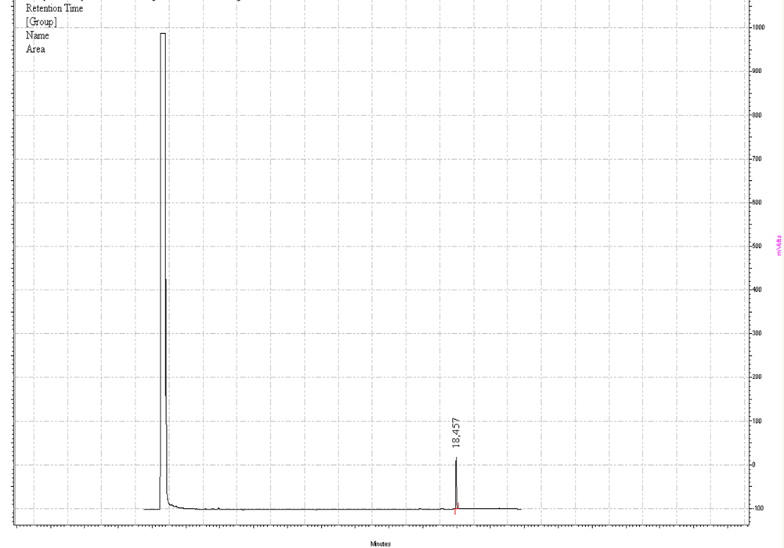


***S* - benzoyl carveol (1j) (18,45min)**


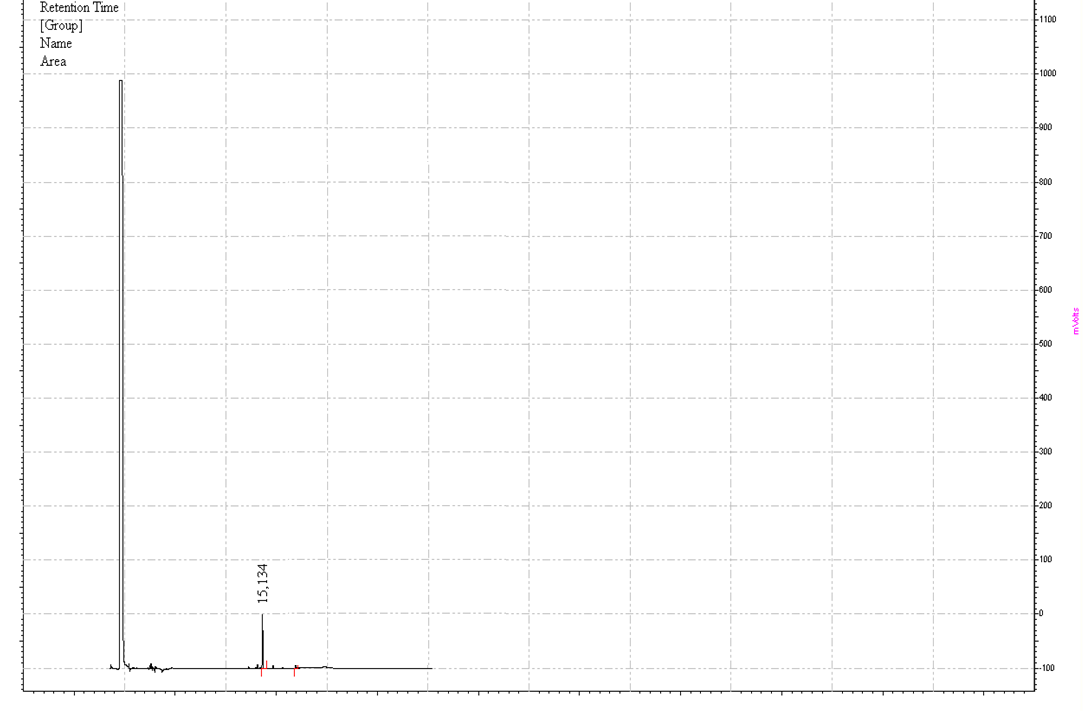


***R* – acetyl carveol (1g) (15,13min)**

**)**


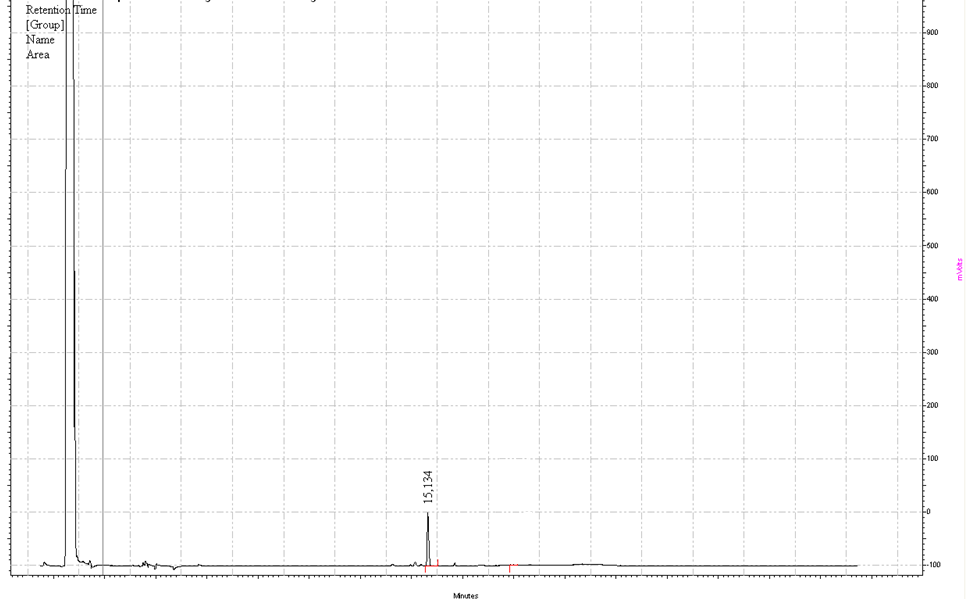


***R* - acetyl carveol (15,13min)**


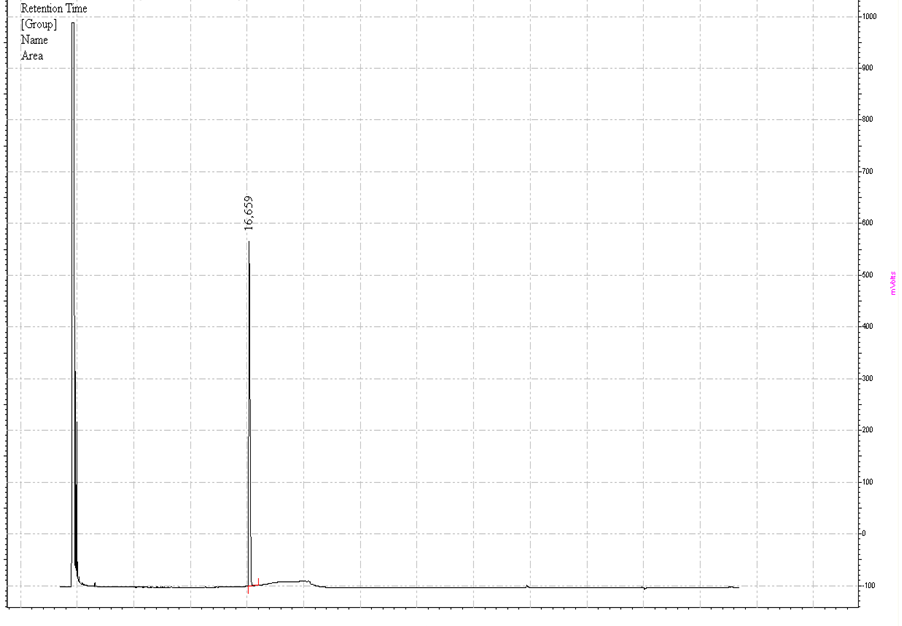


**∆ – 3 – hydroxycarene (2k) (16,65min)**


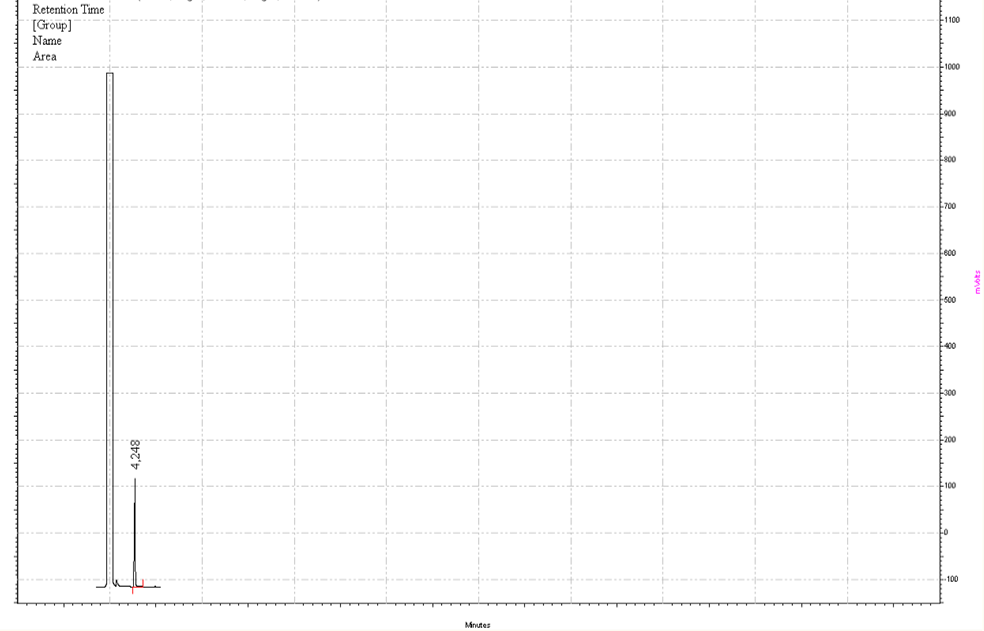


**∆ – 3 – CARENE (1k) (4,24min)**


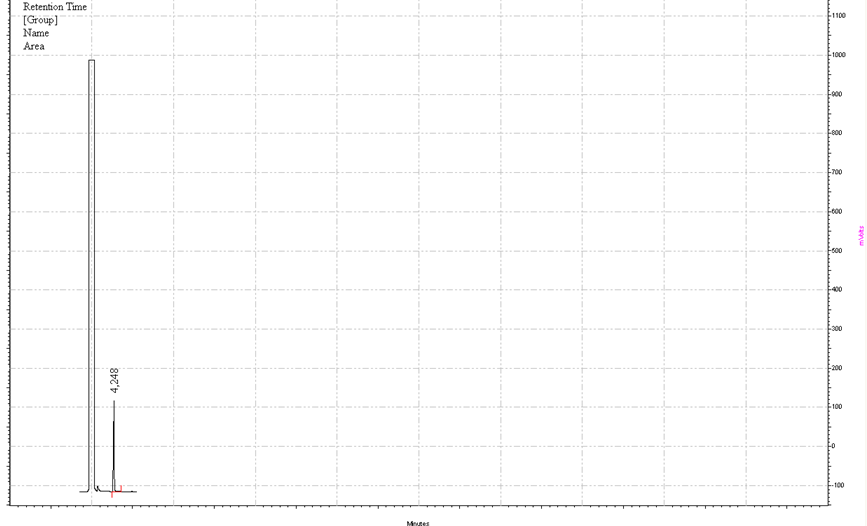


**terpineol (1l) (4,24min)**


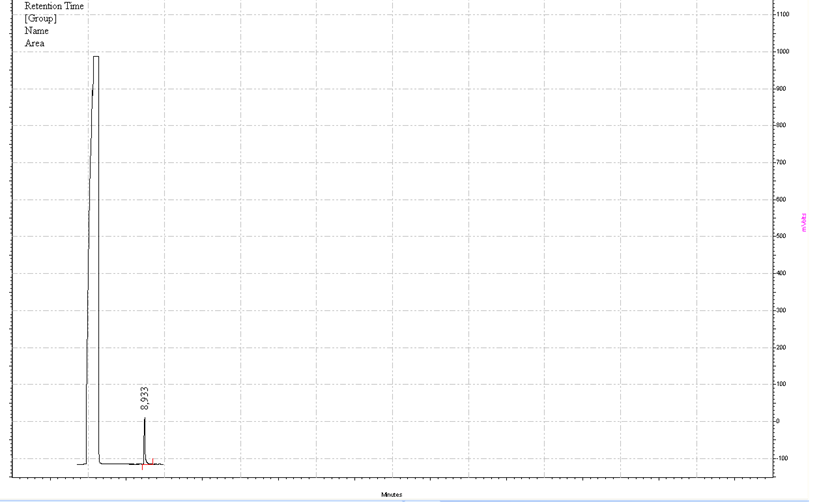


**geraniol (1m) (8,93min)**


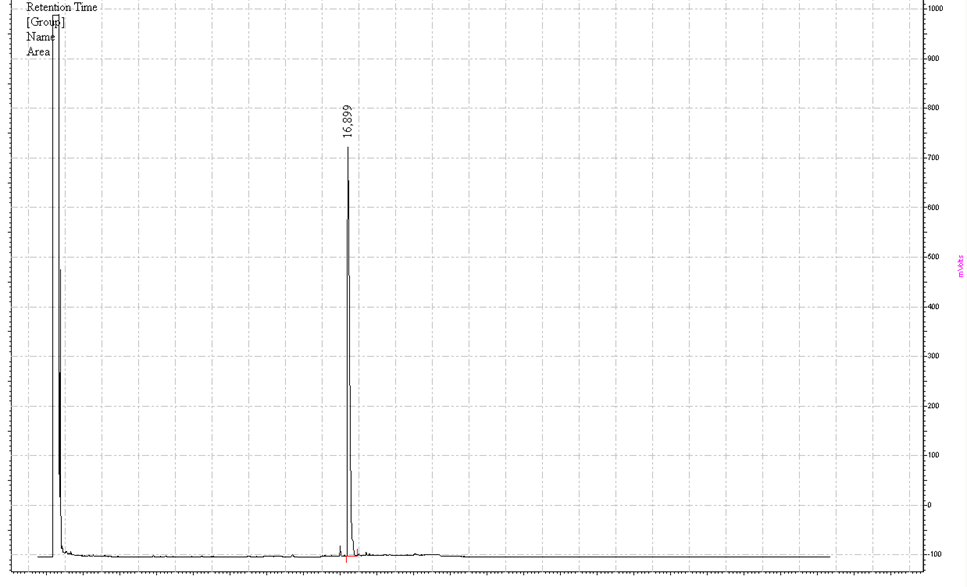


**linalool (1n) (16,89min)**

1. **SDS-PAGE**


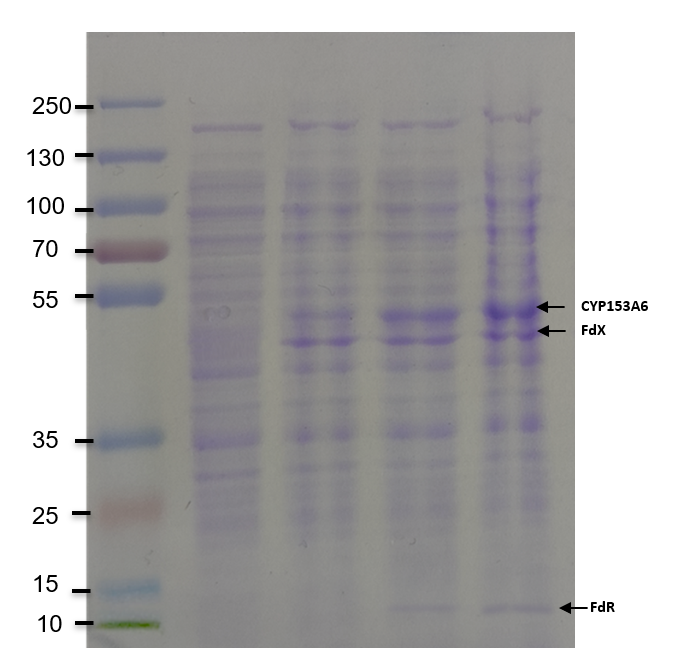


SDS-PAGE on 12% polyacrylamide gel stained with Coomassie blue of cell-free extracts of BL21(DE3)Star *E. coli* strain harbouring pET100-CYP53A6 induced in different media. Cells were harvested after 5 hours of induction (0.5 mM IPTG). Lane 1: Thermofisher prestained protein ladder (#26619); lane 2: BL21(DE3)Star *E. coli* - pET100-CYP53A6 not induced culture; lane 3: BL21(DE3)Star *E. coli* - pET100-CYP53A6 induced in LB medium; lane 3: BL21(DE3)Star *E. coli* - pET100-CYP53A6 induced in TB medium; lane 4: BL21(DE3)Star *E. coli* - pET100-CYP53A6 induced in SB medium. The arrows indicate CYP153A6 (47,7KDa), FdX: ferredoxin (45,4KDa) and FdR: ferredoxin reductase (11,4KDa).
